# 

**Published:** 1866-04

**Authors:** 


### CHICAGO DENTAL SOCIETY.

At the Annual Meeting of the Chicago Dental Society held April 2d 1866 the following officers were elected, viz;

Dr. J. W. Ellis, President.
" M. W. Sherwood, 1st Vice President.
" S. B. Noble, 2d Vice President.
" J. C. Dean, Cor. and Recording Secretary.
" Wm. Albaugh, Treasurer.
" W. A. Stevens, Librarian.
" M. S. Dean,
" W. C Dyer,  } Executive Committee.
" A. J. Harris,

### NORTHERN OHIO DENTAL ASSOCIATION.

The annual meeting will be held in Childs' Hall, at Painesville, on Tuesday, May 1st, 1866, at 1 o'clock, P. M.

### ORDER OF BUSINESS.

1st. Reading of Minutes.
2d. Election of Members.
3d. Election of Officers.
4th. Election of Representatives to the National Association.
5th. Reports of Committees.
6th. Reading of Essays.

### DISCUSSIONS.

1st. The preservation of exposed dental pulps.
2d. The best means of removing exposed dental pulps.
3d. The best method of obtaining accurate impressions and models of the mouth.
4th. Dental Quackery.
5th. Miscellaneous.

Arrangements have been made for first rate hotel accomodations at the Cowles House.

B. F. ROBINSON,
Corresponding Secretary.

Cleveland, April 10, 1866.

### WESTERN NEW YORK DENTAL ASSOCIATION.

### SEMI-ANNUAL CALL.

The Fourth Semi-annual meeting of this Association will be held at Lockport, N. Y., on Tuesday, May 1st, at 10 o'clock A. M., holding for two days.

Operations at the chair will be performed during the meeting, and a session of unusual interest is expected. Members are particularly requested to be present, and all practising dentists desirous of becoming so, are cordially invited to attend.

### SUBJECTS FOR DISCUSSION.

I. Absorption of the alveolar process and loosening of the teeth.—Causes and Treatment.—Drs. F. French, B. T. Whitney.

II. Constitutional effects of diseased teeth and Fangs.—Drs. R. G. Snow, E. H. Danforth.

III. Atmospheric pressure, and its utility.—Drs. L. J. Walter, G. C. Daboll.

IV. Filling teeth.—Drs B. S. Brown, A. P. Southwick.

V. Dental Etiquette.—Drs. S. G. Wood, Geo. B. Snow.

GEO. E. HAYES, President.

GEO. B. SNOW, Secretary.

Buffalo, April 2d, 1866.

### Editorial.

### PROFESSIONAL.

It gives us great pleasure to announce that our long tried friend and much esteemed professional brother. Dr. Wm, H. Goddard, of Louisville Ky., has resumed practice in that city; after a lapse of some seven years, during that time his attention having been given to other pursuits. But no pursuits, no other calling was so congenial to his tastes, as the chosen profession of his younger days.

We know this announcement will be hailed with pleasure by all who know him, and that the brotherhood will gladly welcome him back into the ranks of our profession. He is one of the progressives, and would never be content to occupy a position short of the front rank.

### PERSONAL.

In the last number of the Register, on page 144 in the list of members of the committee to solicit contributions for the Ohio Dental College, appointed by the College Association at its late meeting, the name of Dr. W. H. Shadoan of Louisville Ky., was by oversight ommitted. He was appointed upon that committee, and we know will be one of its active members.

We gladly make this correction which is certainly due Dr. Shadoan.

### MARRIAGE.

Married on Tuesday the 27th of March. Dr. H. A. Smith to Miss Lucy Tomlinson both of this city.

We wish Dr S. and his fair bride all the happiness they are capable of enjoying. We are much in favor of all our freinds being made happy in the same way. Indeed to *all*, of maturity and discretion, we say get married—if it suits you.

In the next number of the Register we shall publish several editorial items that should have appeared in this.

AMERICAN DENTAL ASSOCIATION—Meets annually (this year at Boston,) *Pres.* C. W. SPALDING, of St. Louis, *Rec. Sec.* J. TAFT, of Cincinnati, *Cor. Sec.* L. D. SHEPARD, of Salem, Mass.

*List of Societies represented in the American Dental Association.*

ALBANY DENTAL ASSOCIATION—Meets monthly at Albany. *Pres.* ROBERT NELSON *Rec Sec.* W. F. WINNE, *Cor. Sec.* B. WOOD, of Albany.

BUFFALO DENTAL SOCIETY—Meets monthly at Buffalo. *Pres.* R. G. SNOW, *Rec Sec.* GEO. B. SNOW, of Buffalo.

BROOKLYN DENTAL ASSOCIATION—Meets semi-monthly *Pres.* W. C. PARKS, *Rec. Sec.* W. B. HURD, *Cor. Sec.* W. H. ATKINSON, of New York.

CINCINNATI DENTAL SOCIETY—Meets semi-monthly at Cincinnati. *Pres.* C. H. JAMES, *Rec. Sec.* WM. TAFT.

CENTRAL OHIO DENTAL ASSOCIATION—Meets Semi-annually* on the second Tuesday of May and November. *Pres.* Dr. M. DECAMP, Mansfield, O., *Sec.* A. W. MAXWELL Gallion, O.

CENTRAL STATES DENTAL ASSOCIATION—Meets annually* *Pres.* Dr. W H. MORGAN, *Sec.* W. H. SHADOAN, Louisville, Ky.

CENTRAL NEW YORK DENTAL ASSOCIATION Meets semi-annually. *Pres.* S. B. PALMER, *Rec. Sec.* S. G. MARTIN, *Cor. Sec.* P. HARRIS of Skaneatles.

CHICAGO DENTAL SOCIETY—Meets monthly at Chicago. *Pres.* J. W. ELLIS, *Rec. and* ι *or. Sec.* E. W. SAWYER of Chicago.

CONNECTICUT VALLEY DENTAL ASSOCIATION—Meets semi-annually. second Tuesday of May and October, *Pres.* J. BEALS. *Rec. Sec.* L. D. SHEPHERD, of Salem, Mass.

HUDSON VALLEY DENTAL ASSOCIATION—meets monthly at Troy, N. Y. *Pres.* H. H. YOUNG, *Rec. Sec.* S. J. ANDRES, *Cor. Sec.* S. P. WELCH, of Troy.

INDIANA STATE DENTAL SOCIETY—Meets annually at Indianapolis on the last Tuesday of June. *Pres.* A. M. MOORE, *Rec. and Cor. Sec* JOS. RICHARDSON, of Terre Haute, Ind

IOWA STATE DENTAL SOCIETY—Meets semi-annually Jan. and July. *Pres.* L. C. INGERSOLL, *Rec. Sec.* H. S. CHASE.

LEBANON VALLEY DENTAL ASSOCIATION—*Prest.* W. K. BRENIZER.

MAD RIVER VALLEY DENTAL SOCIETY—Meets quarterly. Next meeting in Dayton, on the first Tuesday in June next. *Prest.*, M. M. OLDHAM, *Sect.*, J. BLAKE.

MICHIGAN DENTAL SOCIETY—Meets annually fourth Tuesday of Jan. at Detroit· *Pres.* J. W. FIELD, *Rec. and Cor. Sec.* G. W. STONE, Albion.

MISSISSIPPI VALLEY DENTAL ASSOCIATION—Meets annually in February at Cincinnati, *Pres.* G. W. KEELY, *Rec. Sec.* WM. TAFT, *Cor Sec.* H. A. SMITH, of Cin.

MISSOURI DENTAL ASSOCIATION—Meets on the first Tuesday of June, 1866, in St Louis. *Pres.* Dr. H. J. MCKELLOPS. St. Louis, *Sec* H. JUDD, St. Louis.

MERRIMAC VALLEY DENTAL SOCIETY—Meets semi annually* first Tuesday of Oct. and May. *Pres.* A. LAWRENCE, *Rec. Sec.* G. A. GERRY, of Lowell.

NASHVILLE DENTAL SOCIETY—Meets on the second Tuesday of each month. *Pres.* W. H. MORGAN, *Sec.* J. C. ROSS

NEW YORK SOCIETY OF DENTAL SURGEONS—Meets semi-monthly in New York, *Pres.* W. H. ALLEN, *Rec. Sec.* C. D. ALLEN, *Cor. Sec.* C. P. FITCH, of New York.

NEW HAVEN DENTAL SOCIETY—Meets on the first Wednesday of each month at New Haven. *Pres.* J. T. METCALF, *Rec. and Cor. Sec.* C. L. SMITH, of New Haven.

NEW LONDON COUNTY DENTAL SOCIETY—Meets monthly.* *Pres.* R. W. BROWNE *Rec. Sec.* WM. ALLENDER.

NORTHERN OHIO DENTAL ASSOCIATION—Meets Semi-annually, (first Tuesday of May and October.) in Cleveland. *Pres.* B. STRICKLAND, *Rec. Sec.* L. BUFFETT, *Cor. Sec* B. F. ROBINSON, Cleveland.

OHIO DENTAL COLLEGE ASSOCIATION—Meets annually in February at Cincinnati. *Pres.* J. H. MCKELLOPS, *Rec. Sec.* J. TAFT, of Cincinnati.

ODONTOGRAPHIC SOCIETY OF PENNSYLVANIA—Meets monthly in Philadelphia, *Pres.* JAS. N. HARRIS, *Rec. Sec.* R. J. HORNER, *Cor. Sec.* J. H. MCQUILLEN, of Phila.

PENNSYLVANIA ASSOCIATION OF DENTAL SURGEONS—Meets monthly in Phila *Pres.* W. W. FOUCHE, *Rec. Sec.* JAMES TRUMAN, *Cor. Sec.* G. W. ELLIS, of Philadelphia

**PITTSBURG DENTAL ASSOCIATION**—Meets monthly, (second Tuesday, in Pittsburg *Pres*. M. E. GILLESPIE, *Rec. Sec*. J. D WHITE.

**PENINSULAR DENTAL SOCIETY**—Meets quarterly.* *Pres*. SAMUEL MARSHALL, *Rec. Sec*. W. G. A. BONWILL, *Cor. Sec*. S. S. NONES.

**WESTERN DENTAL SOCIETY**—Meets annually.* *Pres*. T. P. ABELL, *Rec. Sec*. C. W. SPALDING, *Cor. Sec*. E. A BOGUE.

**ST. LOUIS DENTAL SOCIETY**—Meets monthly at St. Louis.

**WESTERN NEW YORK DENTAL ASSOCIATION**—Meets semi-annually, *(next meeting at Lockport,) *Pres*. GEO. E. HAYES, *Sec* GEO. B. SNOW.

**ST. LOUIS ODONTOLOGICAL SOCIETY**—*Pres*. E. HALE, *Rec. Sec*. G. H. SILVERS, *Cor. Sec*. G. G. SAMUEL.

**AMERICAN DENTAL CONVENTION**—Meets annually first Tuesday of August, (next meeting at New York,) *Pres*. H. E. PEEBLES, *Rec. Sec*. H. A. SMITH. *Cor. Sec*. W. H. ALLEN, of New York.

**Place of meeting fixed by appointment*

### CONTRIBUTORS.

W. H. ATKINSON, M. D., D. D. S.

B. WOOD, M. D.

W. H. SHADOAN.

WM. A. PEASE, D. D. S.

LEON F. HARVEY, M. D.

H. A. SMITH D. D. S.

GEO. B. SNOW, D. D. S.

WM. O. KULP.

A. S. TALBERT, D. D. S.

JOS. RICHARDSON, D. D. S.

A. LAWRENCE.

H. S. CHASE, D. D. S.

R. J. POORE.

GEO. L. PAINE, D. D. S.

A. A. BLOUNT, M. D.

G. A. MILLS.

C. W. SPALDING, D.D.S.

J. DOUGLASS.

J. S. LATIMER, D. D. S.

J. D. ANDERSON, D. D. S,

H. BENEDICT, D D. S.

J. MANSFIELD, D. D. S.

J. F. JOHNSON, D. D. S.

A. M. LESLIE, D. D. S.

GEO. WATT, D. D. S.

J. TAFT, D. D. S.

S. B. PALMER.

F. ABBOTT.

A. BERRY, D. D S.

A. W. MAXWELL.

H. F BISHOP.

JOSEPH S. HILDRETH, M..D

SAM'L JACKSON.

H. T. CRESSINGER.

J. CHESEBROUGH, D. D.

G. W. REDMAN.

### EXCHANGES,

ST. LOUIS MEDICAL AND SURGICAL JOURNAL,
BOSTON MEDICAL AND SURGICAL JOURNAL,
THE PACIFIC MEDICAL AND SURGICAL JOURNAL,
BUFFALO MEDICAL AND SURGICAL JOURNAL,
OHIO MEDICAL AND SURGICAL JOURNAL,
PHILADELPHIA MEDICAL AND SURGICAL JOURNAL,
WESTERN LANCET AND OBSERVER,
CHICAGO MEDICAL JOURNAL,
DENTAL COSMOS,
L' ART DENTAIRE,
THE LONDON DENTAL REVIEW,
BRAITHWAITE'S RETROSPECT,
SAN FRANCISCO MEDICAL PRESS,
THE DENTAL TIMES,
LADIES' REPOSITORY,
HALL'S JOURNAL OF HEALTH,
DEUTSCHE VIERTELJAHRSSCHRIFT,
DENTAL CIRCULAR AND EXAMINER,
CHICAGO MEDICAL EXAMINER.
THE CINCINNATI JOURNAL OF MEDICINE.
MEDICAL RECORDER.
XENIA T RCHLIGHT.
THE U. S. MEDICAL AND SURGICAL JOURNAL.
THE DENTAL QUARTERLY.

### CONTENTS.

OF THE WEST.

*Issued Monthly, at $3 a Year in Advance.*

DEVOTED TO THE INTERESTS OF THE DENTAL PROFESSION.

EDITED BY J. TAFT

The volume commences in January and closes in December.

The Register being issued monthly is always fresh, therefore indispensable to the practitioner who desires to keep posted up with the new inventions and improvements which are daily developed. Neither expense nor effort will be spared to give value and variety to its contents. Articles will be illustrated with well executed engravings whenever they will add to the interest or assist in explanation.

Its extensive circulation and frequency of issue makes it one of the BEST DENTAL ADVERTISING MEDIUMS in the United States.

RATES OF ADVERTISING.

One page, 1 year, $50. Half page, 1 year, $30. Quarter page, or card, 1 year $20.

All advertising bills payable in advance, or at furthest after the issue of the first number containing the advertisement. All transient advertisements to be paid for in advance.

☞ CONTRIBUTIONS SOLICITED.

Address, J. TAFT, or "DENTAL REGISTER," Cincinnati, Ohio.

Business Communications to

J. TAFT, Publisher,
No. 172 Race Street.

